# PECAM-1 drives β-catenin-mediated EndMT via internalization in colon cancer with diabetes mellitus

**DOI:** 10.1186/s12964-023-01193-2

**Published:** 2023-08-14

**Authors:** Qing Wu, Xingxing Du, Jianing Cheng, Xiuying Qi, Huan Liu, Xiaohong Lv, Xieyang Gong, Changxin Shao, Muhong Wang, Luxiao Yue, Xin Yang, Shiyu Li, Yafang Zhang, Xuemei Li, Huike Yang

**Affiliations:** 1 Department of Anatomy, Harbin Medical University, Harbin, China; 2 Department of Humanities Foundation, Heilongjiang Nursing College, Harbin, China; 3 Department of Obstetrics and Gynecology, the Second Affiliated Hospital of Harbin Medical University, Harbin, China; 4 Colorectal Cancer Surgical Ward 2, Harbin Medical University Cancer Hospital, Harbin, China

**Keywords:** Colon cancer, Diabetes mellitus, EndMT, PECAM-1, β-catenin

## Abstract

**Background:**

Diabetes mellitus (DM) is considered to be a risk factor in carcinogenesis and progression, although the biological mechanisms are not well understood. Here we demonstrate that platelet-endothelial cell adhesion molecule 1 (PECAM-1) internalization drives β-catenin-mediated endothelial-mesenchymal transition (EndMT) to link DM to cancer.

**Methods:**

The tumor microenvironment (TME) was investigated for differences between colon cancer with and without DM by mRNA-microarray analysis. The effect of DM on colon cancer was determined in clinical patients and animal models. Furthermore, EndMT, PECAM-1 and Akt/GSK-3β/β-catenin signaling were analyzed under high glucose (HG) and human colon cancer cell (HCCC) supernatant (SN) or coculture conditions by western and immunofluorescence tests.

**Results:**

DM promoted the progression and EndMT occurrence of colon cancer (CC). Regarding the mechanism, DM induced PECAM-1 defection from the cytomembrane, internalization and subsequent accumulation around the cell nucleus in endothelial cells, which promoted β-catenin entry into the nucleus, leading to EndMT occurrence in CC with DM. Additionally, Akt/GSK-3β signaling was enhanced to inhibit the degradation of β-catenin, which regulates the process of EndMT.

**Conclusions:**

PECAM-1 defects and/or internalization are key events for β-catenin-mediated EndMT, which is significantly boosted by enhanced Akt/GSK-3β signaling in the DM-associated TME. This contributes to the mechanism by which DM promotes the carcinogenesis and progression of CC.

**Graphical Abstract:**

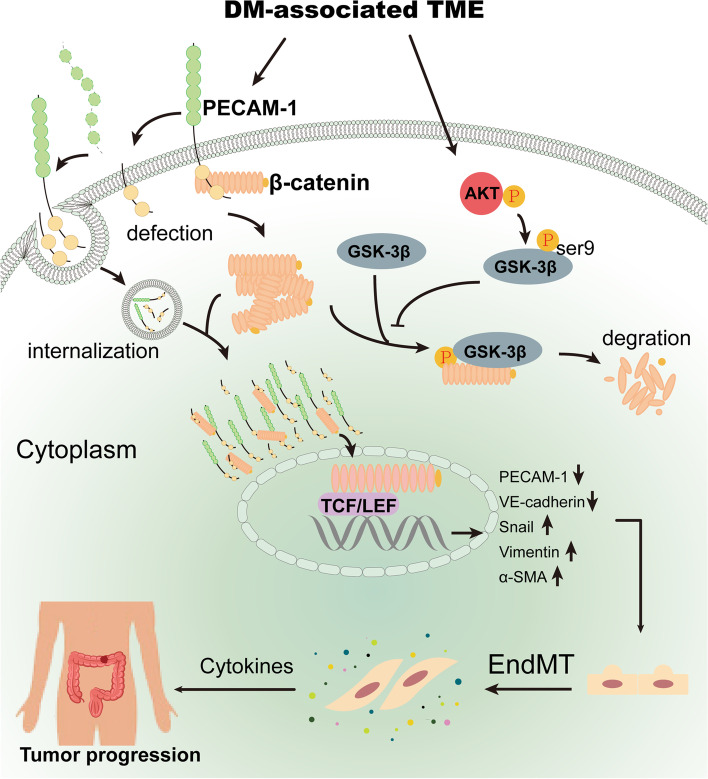

Video Abstract

**Supplementary Information:**

The online version contains supplementary material available at 10.1186/s12964-023-01193-2.

## Background

Increasing evidence suggests that cancer incidence appears to be increased in diabetes mellitus (DM) and even DM is considered to be a risk factor in carcinogenesis and progression [1, 2]. However, the biological mechanisms driving the link between DM and cancer are not well understood. The tumor microenvironment (TME), comprising primarily cancer cells, immune cells, endothelial cells, fibroblasts, and extracellular matrix (ECM), is complex and continuously evolving tissue environment that supports sustained cancer growth, invasion and metastasis [3, 4]. Among them, fibroblasts, defined as interstitial cells of a mesenchymal lineage, namely cancer-associated fibroblasts (CAFs), are implicated closely in cancer development and progression [5]. However, the precise cellular origins and functions of CAFs remain equivocal owing to their heterogeneity in phenotype and function [5]. At present, the putative origins of CAFs include quiescent tissue resident fibroblasts, bone marrow-derived mesenchymal stem cells, endothelial cells, and adipocytes [5]. Researchers have further explored whether EndMT is a key process of endothelial cell transformation into CAFs in the context of cancer [6, 7].

EndMT is an intricate cellular differentiation process in which endothelial cells detach from the cell monolayer and lose cell–cell contacts [8]. During this process, endothelial cells lose their endothelial markers including PECAM-1 (also known as CD31), vascular endothelial-cadherin (VE-cadherin), and von Willebrand Factor (vWF), and acquire mesenchymal markers including vimentin, α-SMA and N-cadherin [8, 9]. Apart from these markers, EndMT also causes a conversion of the cobblestone-like shape of endothelial cells to the elongated spindle shape of mesenchymal cells [9]. EndMT, similar to epithelial–mesenchymal transition (EMT), is an essential physiological process during normal embryologic development and wound healing, and this process can be hijacked to facilitate cancer progression in the context of cancer [9].

We previously found that the mesenchymal cell marker vimentin was strongly expressed, accompanied by a serious disappearance of PECAM-1, in the endothelium in human colon cancer with DM, comparing to that in CC without DM, which implies high-frequency EndMT events in CC with DM. Studies have reported that glucose concentrations can trigger the shift of endothelial cells to mesenchymal cells, which results in myofibroblast formation in diabetes-associated complications [10–12]. Therefore, it is justifiably hypothesized that diabetes-associated EndMT is a possible link between DM and cancer; however, the mechanisms of EndMT in CC with DM remain unclear.

In this study, we determined that PECAM-1 defects and/or internalization and accumulation around the nucleus could promote β-catenin (namely, CTNNB1) translocation to the nucleus, which ultimately leads to EndMT in endothelial cells. During this process, the Akt/GSK-3β signaling pathway was pivotally activated by the DM-associated TME to enhance β-catenin nuclear translocation. Hence, the defect and/or internalization of PECAM-1 in the endothelium with enhanced Akt/GSK-3β signaling is a key mechanism for β-catenin-mediated EndMT in CC with DM.

## Materials and methods

### Cell culture

Human umbilical endothelial cells (HUVECs) (ScienCell, USA) and human colon cancer cells (HCCCs) SW620, SW480, HCT116 and mouse colon cancer cells (MCCCs) MC38 (a gift from Xiaobo Li professor) were used in this study. HUVECs were cultivated with endothelial cell medium (ECM) containing high glucose (HG, 33 mmol/L), normal glucose (NG, 5.5 mmol/L) and 50% supernatant (SN) from HCCCs, respectively [13]. HUVECs were also co-cultivated with SW620 and SW480 cells in a two-dimensional culture system. In addition, basal ECM was supplemented with 5% fetal bovine serum (FBS), 1% endothelial cell growth supplement (ECGS), and 1% penicillin/streptomycin solution (P/S). HCCCs and MCCCs were cultivated in RPMI-1640 medium. HUVECs at passages 3–9 were used in this whole study.

### Animal experiment

C57BL/6 male mice (20–22 g) were purchased from Cyagen Biosciences (Guangzhou). According to previous studies [14, 15], streptozotocin (STZ) (0.1 M, pH 4.5) at the dosage of 100 mg/kg body weight was used to establish DM model by intraperitoneal injection twice. Mice were fed the high-calorie diet containing carbohydrate (45.1%), fat (43.6%) and protein (11.3%). Diabetic mice were used in this study when the fasting blood glucose level is greater than 16.7 mmol/L. MC38 cells (1×10^6^) was injected subcutaneously into the dorsal neck regions of 10 diabetic mice (DM group) and 6 normal mice (non-DM group), respectively [16]. Two weeks after inoculation, visible subcutaneous tumors were detected and subsequently measured every two days for 30 days. In addition, body weight and fasting blood glucose levels of mice were recorded in this experiment.

### Clinical samples

One hundred and ninety-two CC patients, including 108 CCs with DM and 84 CCs, were obtained from Harbin Medical University cancer hospital during January 2015 to December 2021 in this study. Of them, thirty-six pairs of human primary CCs and matched noncancerous adjacent tissues (1.5 cm from the tumor-free margin) were used to protein and mRNA analysis experiments. Each sample was divided into two parts and stored in liquid nitrogen and 4% paraformaldehyde solution at 4 °C, respectively. Clinicopathologic features of recruited patients includes sex, age, DM duration, TNM stage, and survival time.

### Human mRNA array analysis

Ten tissue specimens (5 CCs with DM and 5 CCs) were used for mRNA analysis by Arraystar Human LncRNA Arrays V5. Briefly, total RNA was extracted using TRIzol reagent (Invitrogen, USA) and quantified by a NanoDrop ND-1000. The RNA integrity was assessed using Agilent 2100 Bioanalyzer (Agilent Technologies, USA). Then RNA samples were amplified and transcribed into cDNA, which was subsequently labeled and hybridized to Agilent Array platform. After washing, array scanning was performed with an Agilent DNA Microarray Scanner (Agilent p/n G2505C) and the acquired array images were analyzed with Agilent Feature Extraction software (version 11.0.1.1). Data analysis was performed with using the GeneSpring GX v12.1 software (Agilent Technologies).

### Immunofluorescence test

Tumor tissues fixed with 4% paraformaldehyde were embedded by optimum cutting temperature (OCT) compound and cryosectioned into 6 μm thick sections. Referring to our previous methods [15], these sections were blocked with 5% goat serum followed by the incubation of primary antibody PECAM-1 (1:200) and vimentin (1:200) (table S1a) at 4℃ for overnight and subsequent Alexa Fluor 488-/546- labeled secondary antibody for 1 h at 37℃, and the nuclei were labeled by DAPI. Cells that seeded into the fibronectin (2 μg /cm^2^)-coated coverslips were fixed with 4% paraformaldehyde and performed immunofluorescence test of several markers of EndMT as described above. All images were taken by fluorescence microscopy (Olympus-BX51, Japan) or/and confocal microscopy (Nikon, ECLIPSE Ti-U, Japan).

### Small interference RNA (siRNA) assay

Specific siRNA sequences (table S2) to target PECAM-1 and β-catenin genes (Sangon Biotech, China) were designed to knock down their expressions in this study. When HUVECs reached a 50%–75% confluence, cells were transfected by siRNAs (100 nmol/L) using the X-tremeGENE siRNA Transfection Reagent following the manufacturer’s protocol. Then the siRNA-transfected cells were treated with HG and/or SN for 24–48 h for the EndMT detection. The scramble siRNA was used as a negative control (NC).

### Western blotting analysis

According to the manufacture’s protocol, the extracted whole proteins were determined using Bicinchoninic acid assay (BCA) (Pierce, Rockford, IL, USA). After separating by 10–15% SDS-PAGE gel, proteins were transferred to nitrocellulose filter or PVDF membrane, which followed by the blocking of 5% milk. Then the membrane was probed with primary antibodies (table S1b) overnight at 4 °C and subsequently incubated by horseradish peroxidase (HRP)-conjugated secondary antibodies for 1 h at room temperature. Finally, the antigen–antibody complex was detected by an enhanced chemiluminescence (ECL) system (Amersham Pharmacia Biotech). All experiments were analyzed using Quantity-One software 4.6.2 (Bio-Rad).

### Statistics

All experiments were performed at least thrice and data are presented as the mean ± S.E.M in this study. GraphPad Prism 5.0 (GraphPad Software Inc., USA) and Student’s t-test, one-way analysis of variance (ANOVA), χ^2^ test and Pearson correlation analysis were used for statistical analysis. Differences with *P* values ≤ 0.05 were considered statistically significant.

## Results

### Diabetes mellitus promotes cancer progression and TME remodeling in CC

**Fig. 1:**
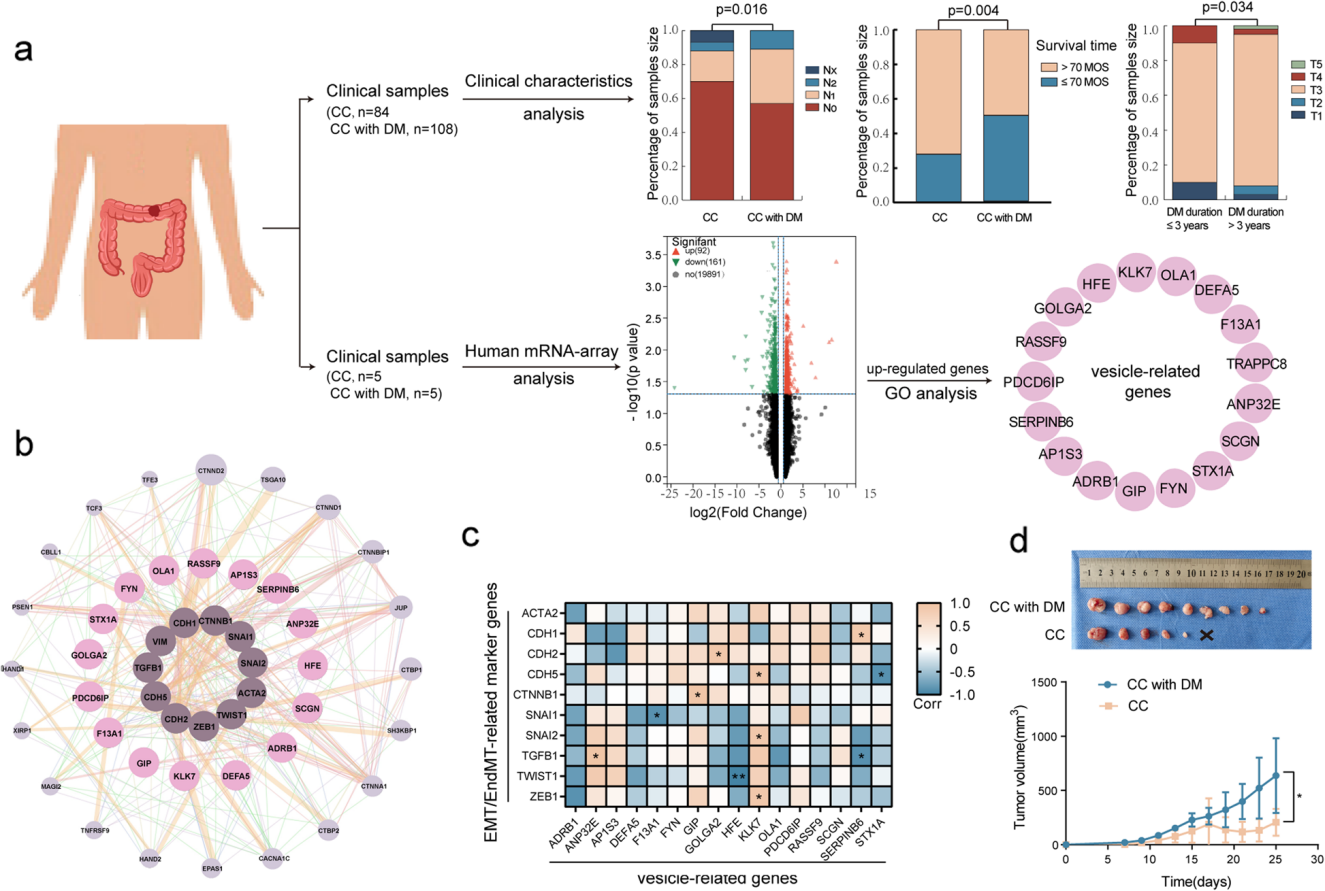
Diabetes mellitus promotes the progression and TME remodeling in CC. **a** Clinical characteristic were analyzed between CC patients with DM and without DM and genes on TME biological diversity were detected by human mRNA-microarray, respectively. **b**, **c** Vesicle-related genes were determined to link to EMT and/or EndMT through Pearson correlation analysis and GENEMANNIA database. **d** Volumes of xenograft were assessed in mice with and without DM. CC: colon cancer, DM: diabetes mellitus, MOS: months. Data are analyzed using unpaired student’s t-test and χ^2^ test. *p < 0.05

To evaluate the effect of DM on CC, we analyzed the clinicopathologic features of CC patients and the xenograft volumes of mice with and without DM. Data showed that DM was positively linked to lymph node metastasis (stage N). CC patients with DM had worse survival rates than CC patients when the survival time was more than 70 months (Fig. 1a and Table 1), but the overall survival was not different between them (Fig. S1a). Furthermore, the group with a long DM duration (> 3 years) had a worse TNM stage in stage T than the group with a short DM duration (≤ 3 years) (Fig. 1a and table S3). In addition, animal experiments indicated that the average volume of xenografts in mice with DM was remarkably larger than that in mice without DM (Fig. 1d and Fig. S1b). These results imply that DM is a risk factor for the malignant progression and poor prognosis of CC.

**Table 1:** Information of clinicopathologic features of CC patients with and without DM

Parameters	Colon cancer (*n* = 84)	Colon cancer with DM (*n* = 108)	Total (*n* = 192)	*P*-value
**Gender**				0.307
Male	49	55	104	
Female	35	53	88	
**Age**				0.300
≤ 55	29	28	57	
> 55	55	80	135	
**T stage**				0.368
T1	1	5	6	
T2	4	5	9	
T3	73	93	166	
T4	2	4	5	
Tx	4	2	6	
**N stage**				0.016^*^
N0	59	61	120	
N1	15	35	50	
N2	4	12	16	
Nx	6	0	6	
**M stage**				
M0	83	108	192	
**Survival time**				0.004^*^
≤ 70 months	16	41	57	
> 70 months	68	67	135	

Several clinicopathologic features such as sex, age, DM duration, TNM stage, and survival time were analyzed between CC patients with DM and CC patients by one-way ANOVA with Tukey’s multiple comparisons test

^*^P < 0.05 indicates a significant association among the variables

Given the role of the TME in tumor progression, we determined the biological diversity of the TME in clinical specimens with and without DM by human mRNA-microarray analysis. Gene Ontology (GO) analysis showed that intracellular vesicles with 17 upregulated genes were significantly enhanced in CC with DM compared to CC without DM (Fig. 1a and Fig. S1c). Among these genes, 16 genes interacted with EMT- or EndMT-related genes (Fig. 1b), and 8 genes were closely associated with their expression (Fig. 1c, d). In addition, biological process analysis revealed that 22.5% (16/71) of the genes were closely related to the cellular response to oxygen levels (Fig. S1e). In view of the vascular endothelium, a key organ sensing and response to oxygen levels, the above data proposed that DM-derived TME alteration or reconstruction might lead to EndMT occurrence.

### HG and the coculture system induced EndMT in vivo and in vitro

**Fig. 2:**
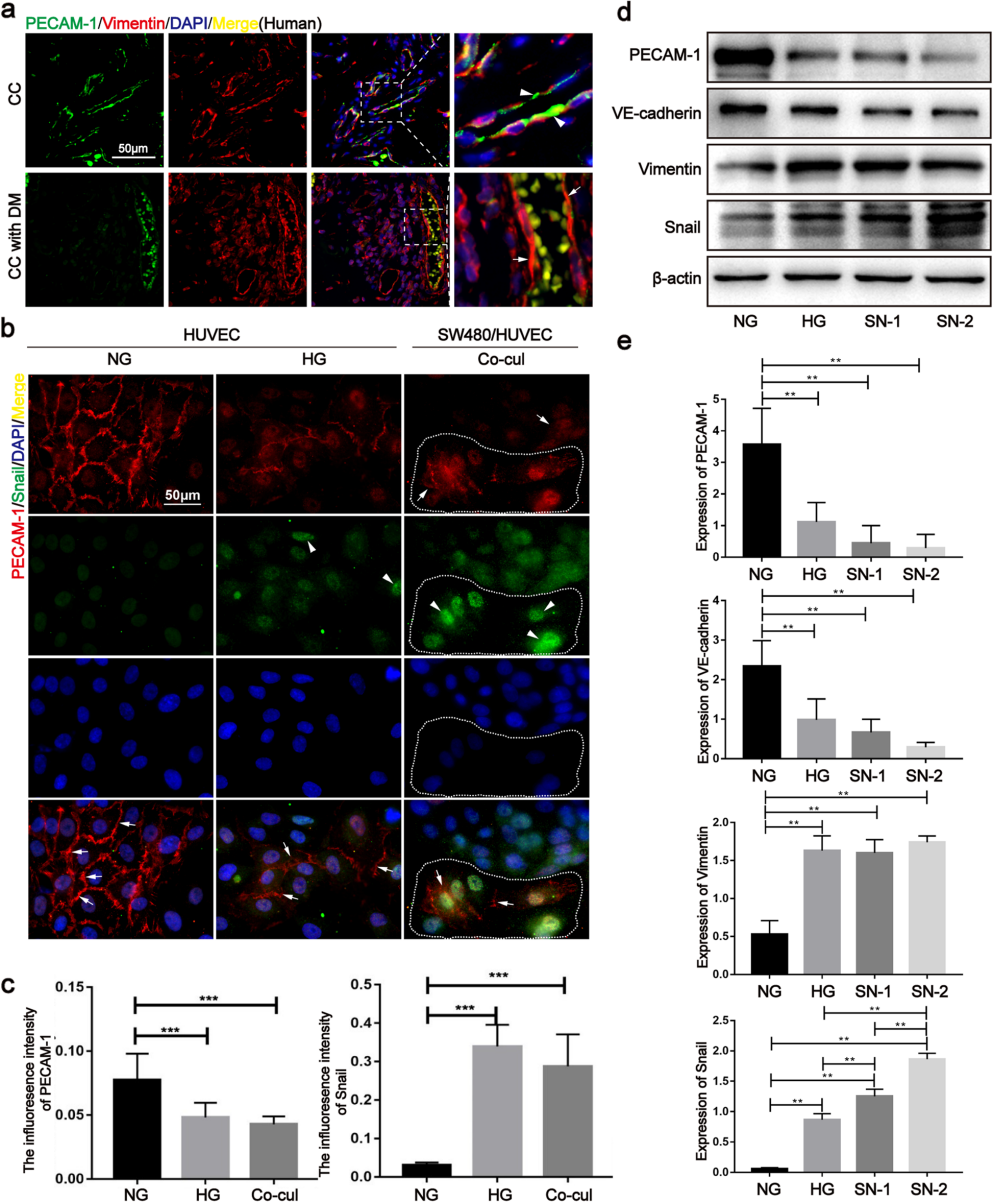
HG and co-culture system induced the EndMT occurrence in vivo and in vitro. **a** Vimentin (arrow) and PECAM-1 (arrow head) were tested in endothelium of CC with and without DM by immunofluorescence. **b**, **c** PECAM-1 (arrow) and snail (arrow head) were analyzed in HG and SW480-HUVEC co-culture system. **d**, **e** EndMT markers were detected under HG and SNs conditions by western blotting. The samples derive from the same experiment and that blots were processed in parallel. NG: normal glucose (5.5 mmol/L), HG: high glucose (33 mmol/L), co-Cul: co-culture, SN-1: supernatant from SW620, SN-2: supernatant from HCT116. Data are means ± S.E.M. and analyzed using unpaired student’s t-test. *p < 0.05, **p < 0.01, ***p < 0.001

Vascular endothelia were detected for EndMT markers in clinical specimens, animal models and in vitro experiments. The results showed that vimentin was enhanced, and PECAM-1 was decreased or lost in CC with DM, but not in CC without DM (Fig. 2a and Fig. S2a). The same result was confirmed in mouse xenograft tissue (Fig. S2b). In the CC and endothelial cell coculture system, PECAM-1 and VE-cadherin were obviously downregulated, but α-SMA and snail were upregulated in endothelial cells (Fig. 2b, c, Fig. S2c and Fig. S3). PECAM-1 was also expressed in CC cells (Fig. 2b, Fig. S2a and Fig. S3a). In addition, we used conditioned medium (CM) containing HG and SNs (SN-1: supernatant from SW620, SN-2: supernatant from HCT116) to treat HUVECs. The results indicated that HG and SNs increased the expression of vimentin and snail and decreased the expression of PECAM-1 and VE-cadherin (Fig. 2d, e). Of note, PECAM-1 was distinctively expressed around the nucleus (namely internalization) under coculture conditions (Fig. 2b and Fig. S3a). In this study, we also found that HG and SNs induced a cell morphological change from a cobblestone-like to elongated spindle shape in HUVECs (Fig. S2d). These data revealed that the DM-related TME facilitates the occurrence of EndMT, where PECAM-1 translocation to the cytoplasm plays a potential role.

### Defects in PECAM-1 promoted EndMT in HUVECs

**Fig. 3:**
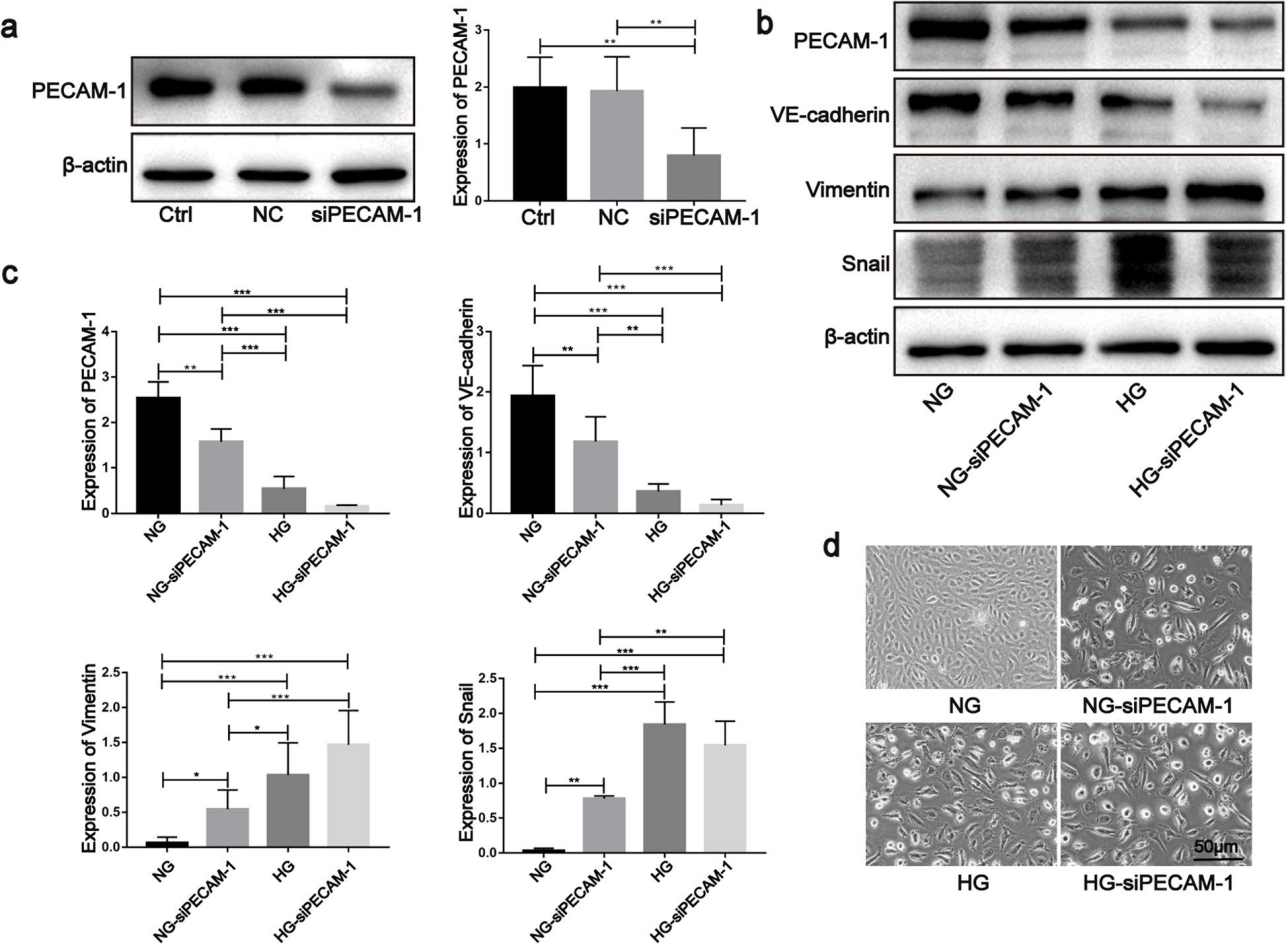
Defection of PECAM-1 promoted the EndMT in HUVECs. **a** PECAM-1 was detected after siRNA treatment by western blotting. **b**, **c** EndMT markers were detected PECAM-1 siRNA treatment by western blotting. **d** Cellular shape was evaluated during EndMT after PECAM-1 siRNA treatment under NG and HG conditions. The samples derive from the same experiment and that blots were processed in parallel. Ctrl: control, NC: negative control, siPECAM-1: PECAM-1 siRNA. Data are means ± S.E.M. and analyzed using one-way ANOVA followed by Tukey’s multiple comparisons test. *p < 0.05, **p < 0.01, ***p < 0.001

Due to PECAM-1 loss, a key property during EndMT occurrence in endothelial cells, we determined the potential role of PECAM-1 in EndMT occurrence by knocking down PECAM-1. The results showed that siRNA could reduce the expression of PECAM-1(Fig. 3a), and its silencing could reduce the expression of VE-cadherin and elevate the expression of vimentin and snail. Then this process was promoted by HG treatment (Fig. 3b, c). In addition, PECAM-1 silencing induced a morphological change in endothelial cells from a cobblestone-like shape to an elongated spindle shape (Fig. 3d). These data imply that PECAM-1 defection is a crucial incident when EndMT occurs under HG conditions.

### β-catenin is a crucial modulator during EndMT in CC with DM

**Fig. 4:**
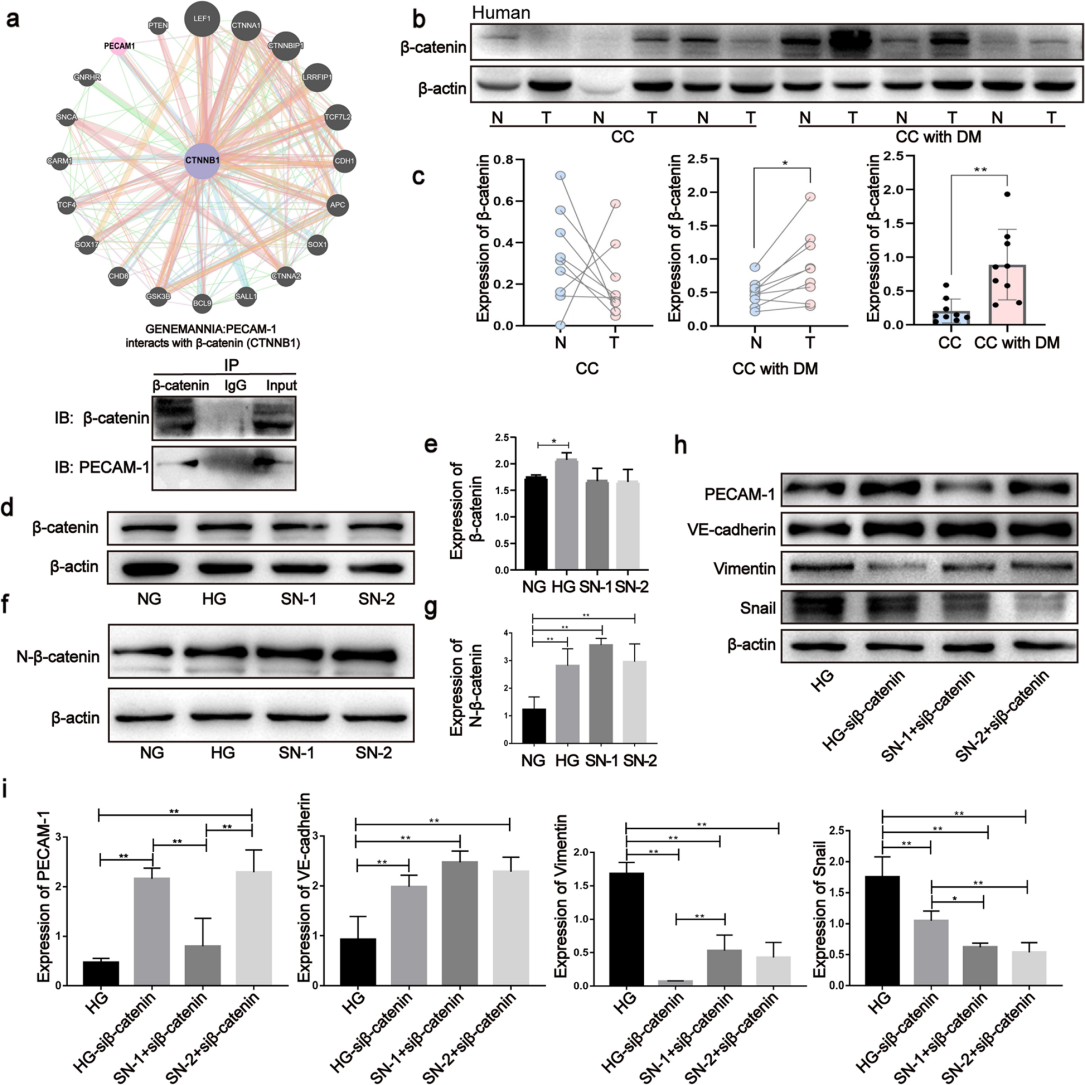
β-catenin is a crucial modulator during the process of EndMT in CC with DM. **a** The interaction of PECAM-1 with β-catenin was assessed through Genemania databases and Co-IP assay. **b**, **c** Expressions of total β-catenin were tested by western blotting in cancerous and noncancerous adjacent tissues of CC with and without DM, respectively. **d-g** Total and nuclear β-catenin were analyzed by western blotting under HG and SNs conditions, respectively. The samples derive from the same experiment and that blots were processed in parallel. Data are means ± S.E.M. and analyzed using unpaired and paired student’s t-test and one-way ANOVA followed by Tukey’s multiple comparisons test. *p < 0.05, **p < 0.01

Furthermore, we found that PECAM-1 interacted with β-catenin through GeneMANIA databases and co-IP assays (Fig. 4a). Data from clinical specimens showed that the expression of β-catenin was not different between cancerous and noncancerous adjacent tissues in CC; however, it was significantly upregulated in cancerous tissues compared to noncancerous adjacent tissues in CC with DM (Fig. 4b, c and Fig. S4a, b). Additional valuable results revealed that CC with DM expressed much more β-catenin than CC (Fig. 4b, c and Fig. S4a, b), which is consistent with results in the mouse model (Fig. S4c, d). Furthermore, in vitro experimental data revealed that HG and SNs could increase the expression of total β-catenin (Fig. 4d, e) and nuclear β-catenin (N-β-catenin) (Fig. 4f, g). In addition, phosphorylated β-catenin at Ser675 (a phosphorylation type to stabilize its location in the nucleus) was increased in clinical specimens and xenograft tissues (Fig. S4a-d). To evaluate the regulatory role of β-catenin in EndMT, we detected the expression of EndMT markers after knocking down β-catenin. The data revealed that β-catenin siRNA suppressed the upregulation of vimentin and snail and the downregulation of PECAM-1 and VE-cadherin (Fig. 4h, i), as well as the mesenchymal cell shape formation induced by HG and SNs (Fig. S4f). These data suggest that β-catenin plays a vital role in EndMT induced by HG and SNs.

### PECAM-1 defects and/or internalization enhanced the nuclear localization of β-catenin

**Fig. 5:**
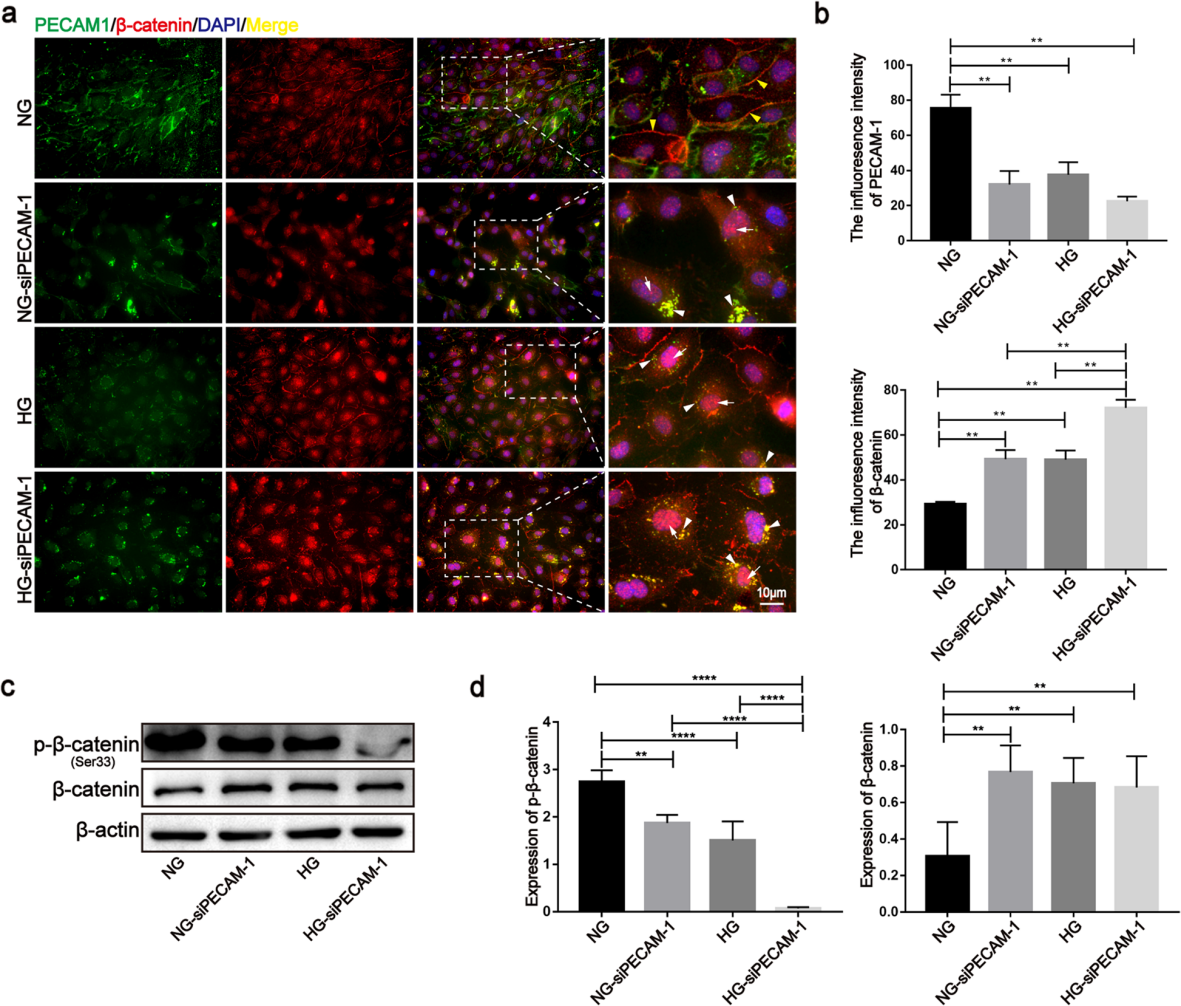
PECAM-1 defection and internalization enhanced the nuclear localization of β-catenin. **a**, **b** Localizations of PECAM-1 and β-catenin were observed after PECAM-1 siRNA treatment by immunofluorescence method under HG and SNs conditions, respectively. White arrows show the nuclear β-catenin; Yellow arrow heads show β-catenin in the membrane; White arrow heads show the colocation of PECAM-1 and β-catenin around the nucleus. **c**, **d** Total and phosphorylated β-catenin (Ser33) (a type to induce its degradation) were evaluated after PECAM-1 siRNA treatment by western blotting under HG and SNs conditions, respectively. The samples derive from the same experiment and that blots were processed in parallel. Data are means ± S.E.M. and analyzed using one-way ANOVA followed by Tukey’s multiple comparisons test. **p < 0.01, ****p < 0.0001

In this study, PECAM-1 knockdown and/or HG conditions led to PECAM-1 defects and/or internalization (Fig. 5a). Furthermore, PECAM-1 silencing decreased phosphorylated β-catenin at Ser33 (a phosphorylation type that results in its degradation) and increased total β-catenin (Fig. 5c, d), as well as promoted its translocation to the nucleus (Fig. 5a, b). In addition to the loss of expression from the cell membrane, PECAM-1 was also found to be coexpressed with β-catenin around the nucleus with an increase in nuclear β-catenin after HG and/or siRNA treatment (Fig. 5a, b), and similar results were found in the SN groups (Fig. S5). These results suggest that PECAM-1 may act as a bridge-like scaffold facilitating β-catenin entry into the nucleus when it transfers from the membrane to the periphery of the nucleus under coculture and HG conditions.

### Akt/GSK-3β signaling was activated to suppress β-catenin degradation in CC with DM

**Fig. 6:**
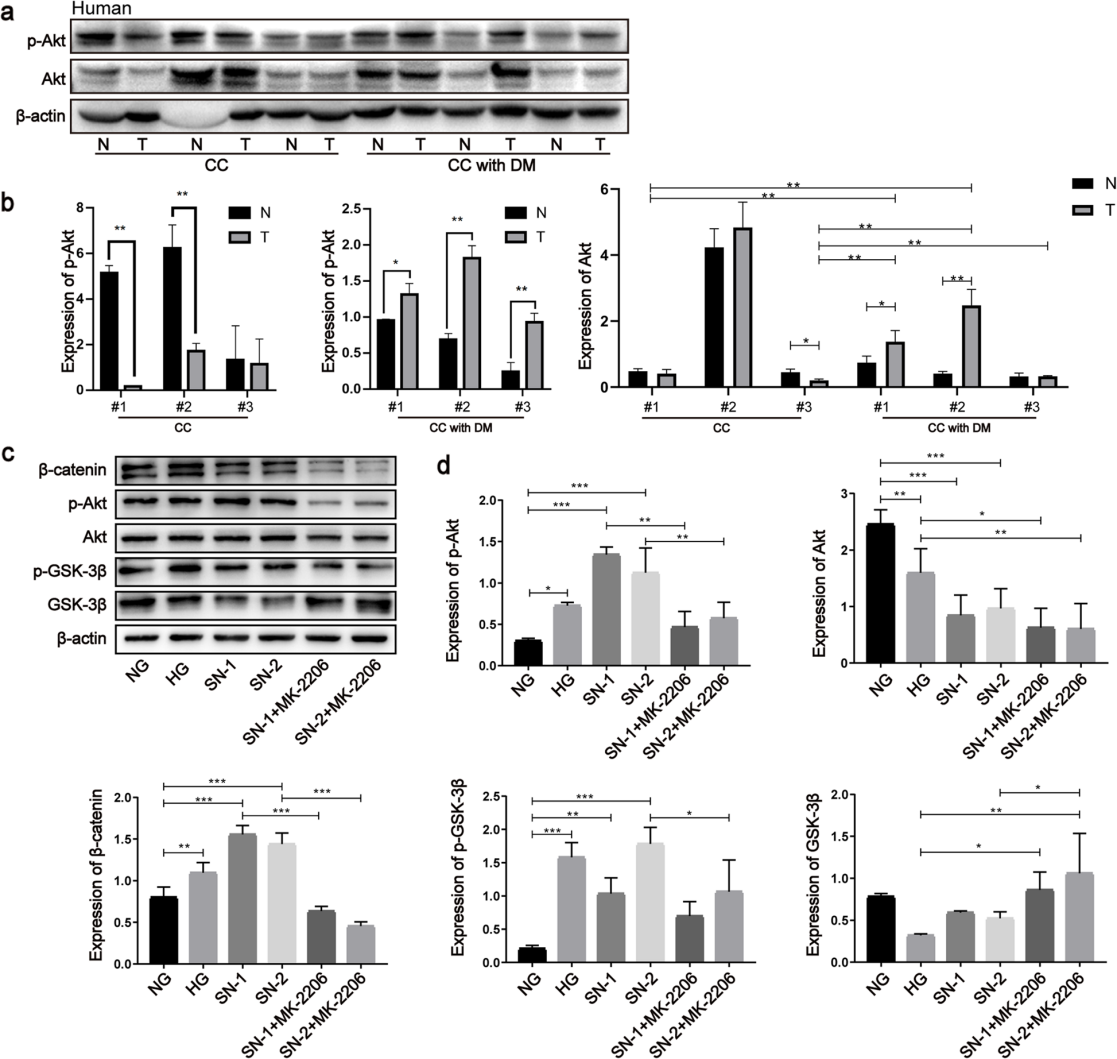
Akt/GSK-3β signaling was activated to suppress the β-catenin degradation in CC with DM. **a**, **b** Total and phosphorylated Akt (Ser473) were evaluated by western blotting in cancerous and noncancerous adjacent tissues of CC with and without DM, respectively. **c**, **d** β-catenin, the phosphorylation levels of Akt (Ser473) and GSK-3β (Ser9) were detected by western blotting under HG and SNs conditions and after the treatment of Akt inhibitor MK-2206. The samples derive from the same experiment and that blots were processed in parallel. Data are means ± S.E.M. and analyzed using one-way ANOVA followed by Tukey’s multiple comparisons test. *p < 0.05, **p < 0.01, ***p < 0.001

In parallel with the expression of β-catenin, the phosphorylation level of Akt (Ser473) was elevated in cancerous tissues compared to that in noncancerous adjacent tissues in CC with DM; however, the opposite was observed in CC (Fig. 6a, b). Phosphorylated Akt was enhanced in xenografts with DM compared to xenografts without DM (Fig. S6a, b). Additionally, HG and SNs significantly enhanced the phosphorylation levels of Akt (Ser473) and GSK-3β (Ser9) in HUVECs (Fig. 6c, d). Furthermore, MK-2206, an inhibitor of Akt, restrained GSK-3β phosphorylation and β-catenin accumulation induced by HG and SNs (Fig. 6c, d). Dishevelled (Dvl), an inhibitor of the β-catenin destruction complex (namely, the APC/Axin/GSK-3β complex), was upregulated in the CC with DM group compared with the CC group (Fig. S6c, d). These data indicate that HG and SN mediums affect the expression of β-catenin by regulating the activation of Akt, the subsequent phosphorylation of GSK-3β and the formation of the β-catenin degradation complex. Additionally, further research showed that PECAM-1 silencing also promoted phosphorylated Akt (Ser473) and GSK-3β (Ser9) (Fig. S6e, f), suggesting that PECAM-1 could play a regulatory role in the activity of Akt.

## Discussion

In addition to traditional complications such as cardiovascular disorders, cancer has been increasingly considered an emerging complication of DM in recent years [17]. Cancer is becoming the leading cause of death from DM due to improvements in management and a decline in the incidence of traditional complications [17]. Our research revealed that DM has a negative effect on CC patients in terms of survival time and TNM stage (T and N stages). Previous studies have proposed potential etiological factors to link DM and cancer, including hyperinsulinemia, hyperglycemia, inflammation, and increased reactive oxygen species (ROS) [17, 18], but the mechanism remains unclear. In this study, we illustrated that PECAM-1 drives β-catenin-mediated EndMT via internalization in CCs with DM, a crucial mechanism linking DM to cancer.

Our studies determined that the TME was remodeled in CC with DM and was dramatically involved in intracellular vesicle incidents. Bioinformatics analysis revealed that these vesicle-related genes were closely associated with EMT or EndMT. As expected, we confirmed a high-frequency occurrence of EndMT in CC with DM compared to that in CC, and the same results were confirmed in both animal models and cultured cells. These data suggest that DM induces TME reconstruction in CC with DM, which is involved in EndMT occurrence. In addition, many studies have shown that EndMT is a key mechanism in DM-associated fibrosis, such as diabetic kidney disease, cardiac fibrogenesis, and pulmonary fibrosis [19–21]. Therefore, DM is considered a crucial stimulator of EndMT among many biological factors in CC with DM. However, how DM promotes EndMT occurrence is unknown. Previous studies revealed that PECAM-1 decrease was a common phenomenon of damaged endothelial cells induced by DM [15, 22, 23]. In our research, in addition to the loss from the cell membrane, PECAM-1 was also found to transfer into the cytoplasm and accumulate around the nucleus, namely PECAM-1 was partially internalized, under HG and coculture conditions. In addition, PECAM-1 silencing induced EndMT in HUVECs. These results suggested that PECAM-1 might be a key controller that modulates EndMT when it is in the cytoplasm.

To elucidate the possible mechanism, we analyzed the potential interaction targets of PECAM-1 through Genemania databases and Co-IP experiments. Our data reveal that PECAM-1 interacts with β-catenin. Previous studies reported that PECAM-1 mediates β-catenin localization to the cell membrane [24, 25]; however, our findings showed that PECAM-1 was coexpressed with β-catenin around the nucleus when PECAM-1 was located in the cytoplasm. Furthermore, cancer-endothelial cell coculture and HG conditions dramatically promoted their colocalization around the nucleus, which was accompanied by elevated expression of β-catenin in the nucleus. These results suggest that PECAM-1 in the cytoplasm may act as a bridge-like structure to facilitate β-catenin entry into the nucleus. This might be a novel regulatory mechanism by which β-catenin enters the nucleus through the internalization of PECAM-1, and it will be worth focusing on in the future. In addition, given this scaffold role for β-catenin localization to the membrane, we disrupted PECAM-1 expression by siRNA to release β-catenin from the membrane. The results revealed that PECAM-1 knockdown increased the cytoplasmic level and nuclear expression of β-catenin, suggesting that it might be another crucial cause for β-catenin to accumulate abundantly in the cytoplasm under coculture and HG conditions. In this study, PECAM-1 was also expressed in CC cells, which maybe modulate cancer cell growth and metastasis, being parallel with the report [26].

As a well-known transcription factor, β-catenin is expressed in various cells and controls the transcription of many genes related to diverse biological processes. Of note, our study revealed a valuable expression profile of β-catenin in CC with and without DM. In detail, total β-catenin was significantly increased in CC with DM compared to that in CC, and it was obviously higher in cancerous tissues than in noncancerous adjacent tissues in CC with DM, but there was no difference in CC. Additionally, in vitro, HG and SNs increased the expression of total β-catenin, including nuclear β-catenin, in HUVECs. Knocking down β-catenin rescued the enhancements of EndMT-associated markers and alteration of cell shape induced by HG and SNs. Therefore, β-catenin is considered a vital modulator of EndMT in CC with DM. These results proposed that diabetes-induced PECAM-1 loss and/or internalization facilitated β-catenin accumulation in the cytoplasm and subsequently entered the nucleus, which ultimately promoted EndMT in the endothelium.

β-catenin is a central member of the canonical Wnt/β-catenin signaling pathway which is widely studied in the context of diverse diseases, such as cancer, cardiovascular disease, and age-associated disorders [27, 28]. As a substrate of glycogen synthase kinase 3β (GSK-3β), β-catenin is phosphorylated by GSK-3β and subsequently degraded by the proteasome through ubiquitination. In contrast, phosphorylated GSK-3β (Ser9) can inhibit the degradation of β-catenin and in turn promote its translocation to the nucleus [29]. In addition, Dvl is a vital regulator that disrupts the degradation of β-catenin in the canonical Wnt signaling pathway [30]. In this study, we determined that the phosphorylation of GSK-3β (Ser9) was enhanced by HG and SNs and that Dvl was elevated in CC tissues with DM. These data indicate that the dysfunction of GSK-3β is another vital cause of β-catenin accumulation in the cytoplasm and entry into the nucleus during β-catenin-mediated EndMT. As a crucial glycogen synthase regulator in glucose metabolism, GSK-3β activity is regulated by several kinases such as Akt (protein kinase B, PKB), extracellular signal-regulated kinase (ERK), PKA, and PKC [31]. Intriguingly, our data showed that the activity of Akt in cancerous tissues was significantly elevated compared to that in noncancerous adjacent tissues in CC with DM, but not in CC. The enhanced activity of Akt was also confirmed in diabetic mice with CCs and in vitro studies. These results propose that DM may be a key factor for the activation of Akt, which is in parallel with previous studies showing that PI3K/Akt signaling is activated in diverse diseases, especially cancers and DM [32]. Furthermore, the inhibition of Akt by MK-2206 suppressed the phosphorylation of GSK-3β (Ser9) and β-catenin upregulation, suggesting that the Akt/GSK-3β axis is an additional vital signaling pathway that regulates the localization of β-catenin.

**Fig. 7:**
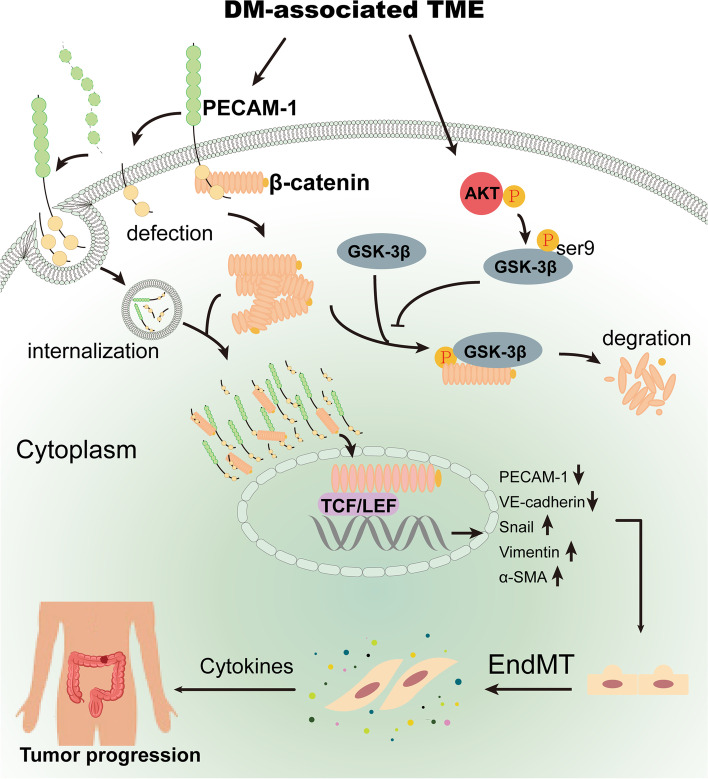
the mechanism that PECAM-1 drives β-catenin-mediated EndMT via internalization in colon cancer with diabetes mellitus

In summary, this study illustrates a novel mechanism by which PECAM-1 in the cytoplasm drives β-catenin-mediated EndMT via internalization, which is promoted by Akt/GSK-3β signaling in CC with DM (Fig. 7). In brief, (1) DM-derived TME remodeling induces PECAM-1 defects and/or internalization in endothelial cells, which facilitates β-catenin release from the cell membrane, accumulation in the cytoplasm and subsequent entry into the nucleus. (2) TME remodeling significantly activates the Akt/GSK-3β signaling pathway to inhibit β-catenin degradation, which leads to the accumulation of β-catenin in the cytoplasm and its translocation to the nucleus. These events ultimately promote EndMT occurrence and in turn stimulate the progression of CC with DM.

### Supplementary Information

**Additional file 1.** **Figure S1.** Diabetes mellitus promotes the progression and TME remodeling in CC. **Figure S2.** HG and co-culture system induced the EndMT occurrence in vivo and in vitro. **Figure S3.** HG and co-culture system induced the EndMT occurrence in vitro. **Figure S4.** β-catenin is a crucial modulator during the process of EndMT in CC with DM. **Figure S5.** SNs induces the alteration of PECAM-1 and β-catenin in localization. **Figure S6.** Expressions of Akt, GSK-3β and Dvl were detected by western blotting. **Table S1.** Primary antibodies in this study. **Table S2.** Sequences of siRNA in this study. **Table S3.** Information of clinicopathologic features of CC patients with and without DM.

## Data Availability

The datasets used and/or analyzed during the current study are available from the corresponding author on reasonable request.

## Abbreviations

DM: Diabetes mellitus
PECAM-1: Platelet-endothelial cell adhesion molecule 1
EndMT: Endothelial-mesenchymal transition
TME: Tumor microenvironment
HG: High glucose
HCCCs: Human colon cancer cells
CC: Colon cancer
ECM: Extracellular matrix
CAFs: Cancer-associated fibroblasts
VE-cadherin: Vascular endothelial-cadherin
vwf: Von Willebrand Factor
EMT: Epithelial–mesenchymal transition
MCCCs: Mouse colon cancer cells
ROS: Reactive oxygen species
