# Supplementary material for: PECAM-1 drives β-catenin-mediated EndMT via internalization in colon cancer with diabetes mellitus
Source: Cell Commun Signal. 2023 Aug 14;21:203. doi: 10.1186/s12964-023-01193-2 (PMC10426208; doi:10.1186/s12964-023-01193-2)
Supplement: Supplementary file 2 — Additional file 1. Figure S1. Diabetes mellitus promotes the progression and TME remodeling in CC. Figure S2. HG and co-culture system induced the EndMT occurrence in vivo and in vitro. Figure S3. HG and co-culture system induced the EndMT occurrence in vitro. Figure S4. β-catenin is a crucial modulator during the process of EndMT in CC with DM. Figure S5. SNs induces the alteration of PECAM-1 and β-catenin in localization. Figure S6. Expressions of Akt, GSK-3β and Dvl were detected by western blotting. Table S1. Primary antibodies in this study. Table S2. Sequences of siRNA in this study. Table S3. Information of clinicopathologic features of CC patients with and without DM. [file 12964_2023_1193_MOESM1_ESM.docx]

**Supplementary information**

PECAM-1 drives β-catenin-mediated EndMT via internalization in colon cancer with diabetes mellitus

Qing Wu^1 *^, Xingxing Du^1 *^, Jianing Cheng^1 *^, Xiuying Qi^1^, Huan Liu^2^, Xiaohong Lv^1^, Xieyang Gong^3^, Changxin Shao^3^, Muhong Wang^4^, Luxiao Yue^1^, Xin Yang^1^, Shiyu Li^1^, Yafang Zhang^1^🖂, Xuemei Li^1^🖂 and Huike Yang^1^🖂

1. Department of Anatomy, Harbin Medical University, Harbin, China.

2. Department of Humanities Foundation, Heilongjiang Nursing College, Harbin, China.

3. Department of Obstetrics and Gynecology, the Second Affiliated Hospital of Harbin Medical University, Harbin, China.

4. Colorectal Cancer Surgical Ward 2, Harbin Medical University Cancer Hospital, Harbin, China.

* These authors contributed equally to this work

Corresponding authors: Huike Yang, Xuemei Li, Yafang Zhang

🖂 Email: huikeyang@hrbmu.edu.cn; xuemeili@hrbmu.edu.cn; yafangzhang2008@aliyun.com.

Huike Yang, ORCID: 0000-0003-3876-4914

**Abstract**

**BACKGROUND:** Diabetes mellitus (DM) is considered to be a risk factor in carcinogenesis and

progression, although the biological mechanisms are not well understood. Here we demonstrate that platelet-endothelial cell adhesion molecule 1 (PECAM-1) internalization drives β-catenin-mediated endothelial-mesenchymal transition (EndMT) to link DM to cancer.

**METHODS:** The tumor microenvironment (TME) was investigated for differences between colon cancer with and without DM by mRNA-microarray analysis. The effect of DM on colon cancer was determined in clinical patients and animal models. Furthermore, EndMT, PECAM-1 and Akt/GSK-3β/β-catenin signaling were analyzed under high glucose (HG) and human colon cancer cell (HCCC) supernatant (SN) or coculture conditions by western and immunofluorescence tests.

**RESULTS:** DM promoted the progression and EndMT occurrence of CC. Regarding the mechanism, DM induced PECAM-1 defection from the cytomembrane, internalization and subsequent accumulation around the cell nucleus in endothelial cells, which promoted β-catenin entry into the nucleus, leading to EndMT occurrence in CC with DM. Additionally, Akt/GSK-3β signaling was enhanced to inhibit the degradation of β-catenin, which regulates the process of EndMT.

**CONCLUSIONS:** PECAM-1 defects and/or internalization are key events forβ-catenin-mediated EndMT, which is significantly boosted by enhanced Akt/GSK-3β signaling in the DM-associated TME. This contributes to the mechanism by which DM promotes the carcinogenesis and progression of colon cancer.

**KEYWORDS:** colon cancer, diabetes mellitus, EndMT, PECAM-1, β-catenin

**SUPPLEMANTARY RESULTS**

**
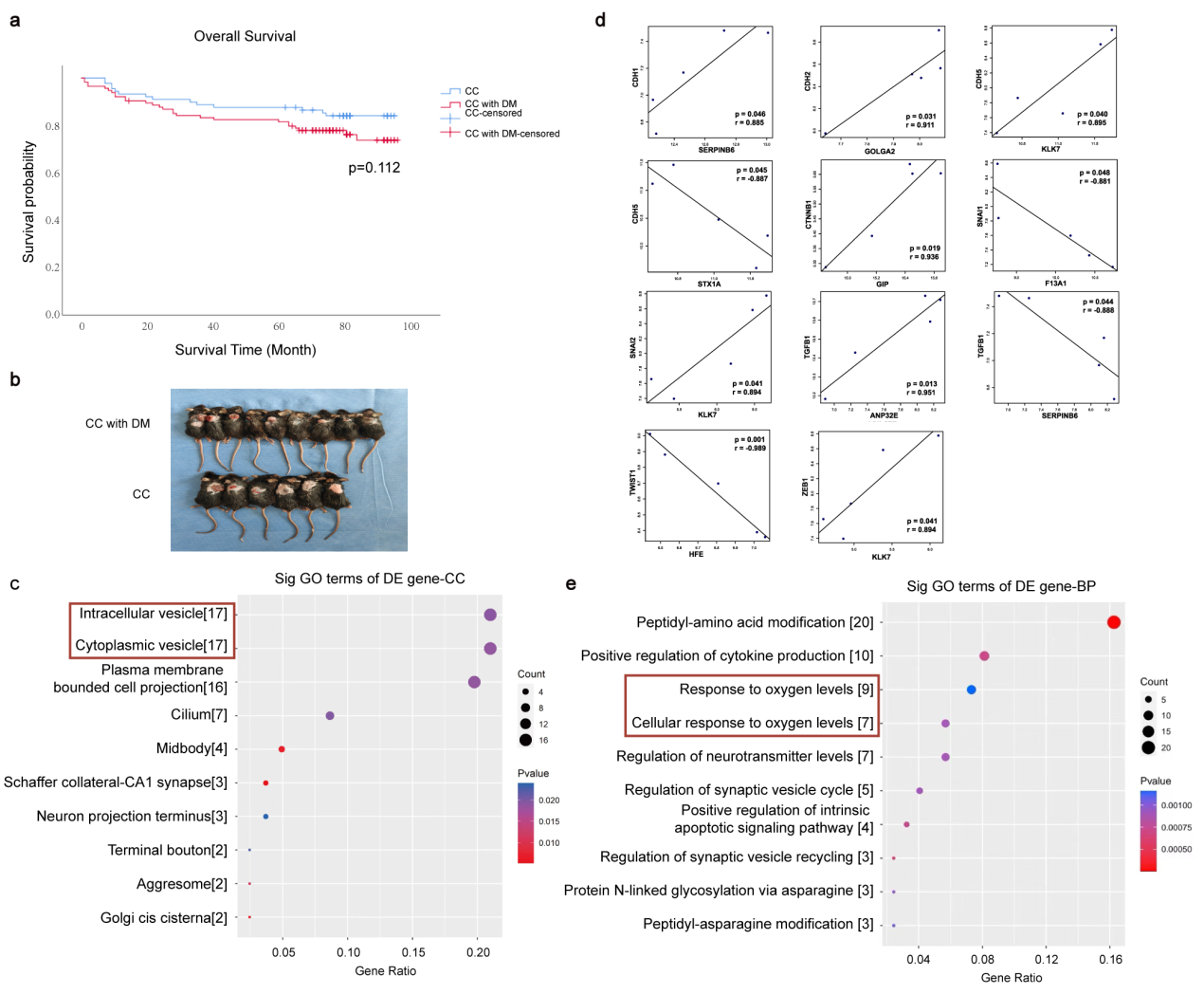
**

**Figure S1 Diabetes mellitus promotes the progression and TME remodeling in CC**

**a** Overall survival was evaluated between CC patients with and without DM. **b** Ten diabetic mice with CC xenograft and 6 mice with CC xenograft were used I this study. **c, e** Data from human mRNA-microarray analysis were assessed by gene ontology (GO) and showed two enrichments that the alteration of cellular response to oxygen level in biological process analysis (BP) and the formation of intracellular vesicle in cellular component (CC) processes, respectively. **d** Pearson correlation analysis showed that 8 vesicle-related genes were associated closely with EMT or EndMT marker genes. Data are analyzed using Kaplan-Meier test.

**
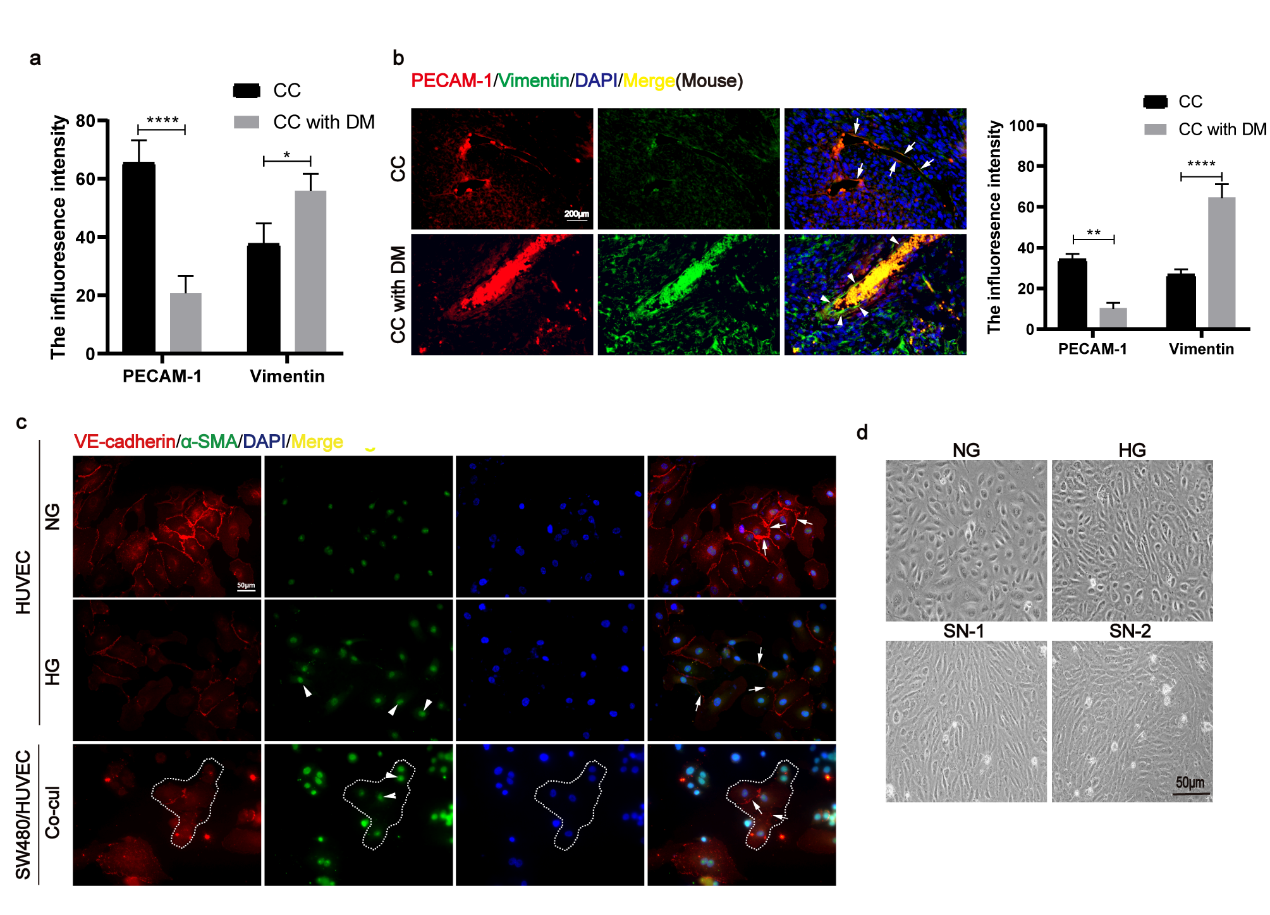
**

**Figure S2 HG and co-culture system induced the EndMT occurrence in vivo and in vitro**

**a.** Quantification of PECAM-1 and Vimentin of figure 2a. **b** PECAM-1 (arrow) and vimentin (arrow head) of endothelium were tested in xenograft tissues of mice with and without DM by immunofluorescence assay. **c** VE-cadherin (arrow) and α-SMA (arrow head) of HUVECs were analyzed in HG and SW480-HUVEC co-culture system. **d** HG and SNs induced a cell morphological change of cobblestone-like to elongated spindle shape in HUVECs.


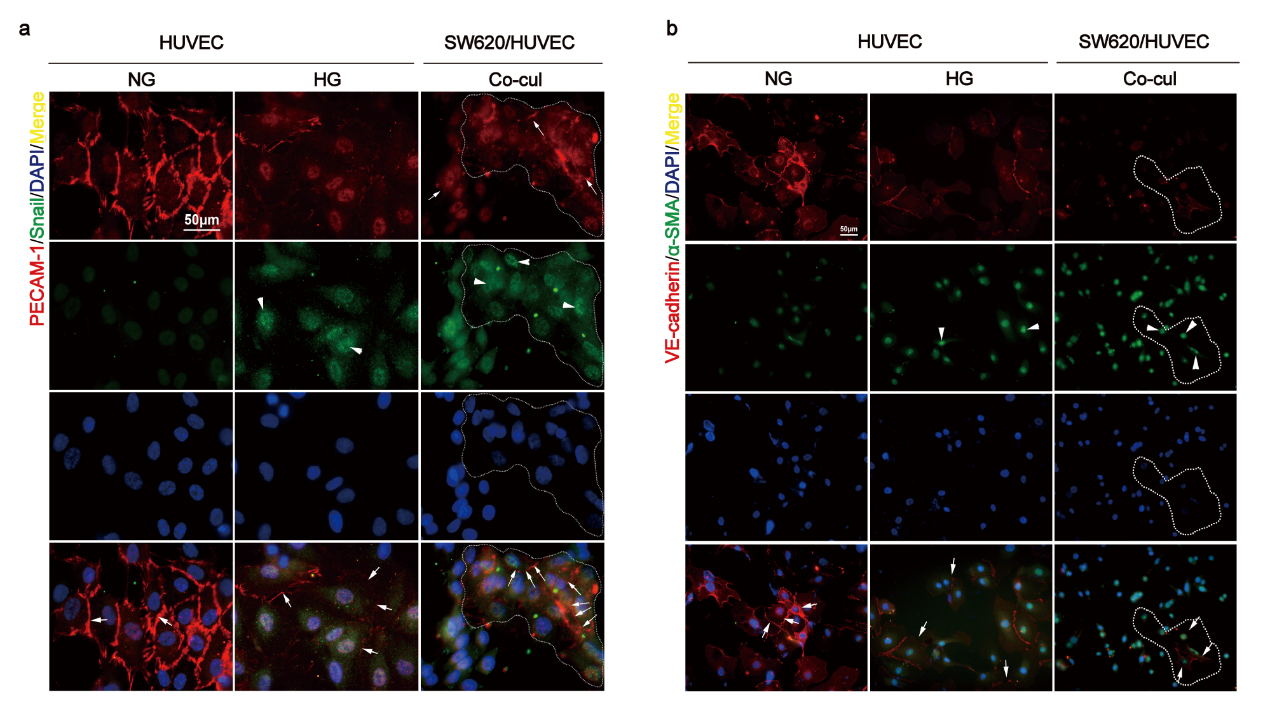


**Figure S3 HG and co-culture system induced the EndMT occurrence in vitro**

**a** PECAM-1 (arrow) and snail (arrow head) were analyzed in HG and SW620-HUVEC co-culture system. **b** VE-cadherin (arrow) and α-SMA (arrow head) of HUVECs were analyzed in HG and SW620-HUVEC co-culture system.


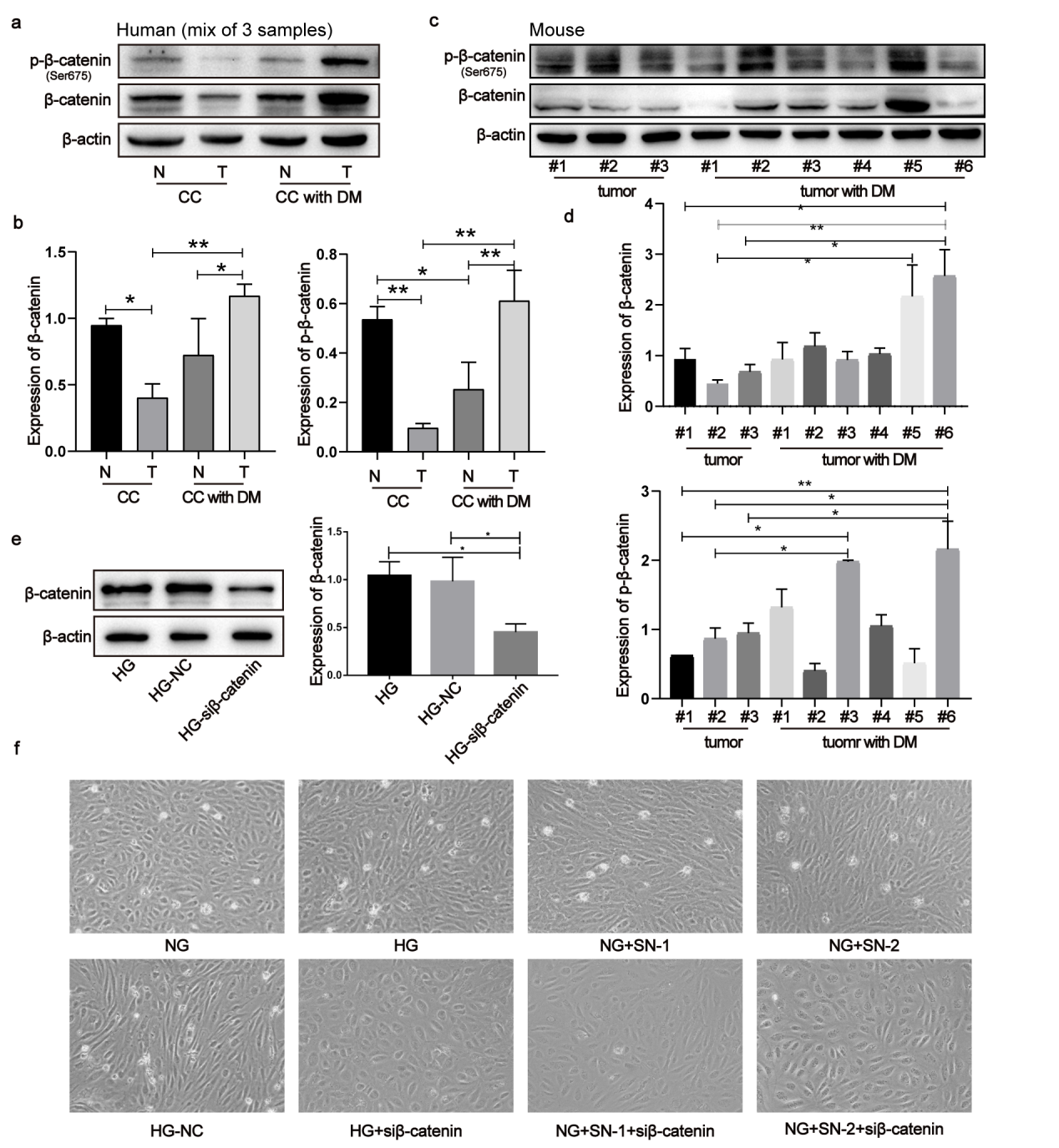


**Figure S4 β-catenin is a crucial modulator during the process of EndMT in CC with DM**

**a, b** Expressions of phosphorylated and total β-catenin were tested by western blotting in cancerous and noncancerous adjacent mixed tissues of CC with and without DM, respectively. **c, d** Expressions of phosphorylated and total β-catenin were tested by western blotting in xenograft tissues of CC with and without DM. **e** The total β-catenin was detected after siRNA treatment by western blotting. **f** Cellular shape was evaluated during EndMT after β-catenin siRNA treatment under NG and HG conditions. The samples derive from the same experiment and that blots were processed in parallel. Data are means ± S.E.M. and analyzed using one-way ANOVA followed by Tukey’s multiple comparisons test. **p*<0.05, ***p*<0.01.


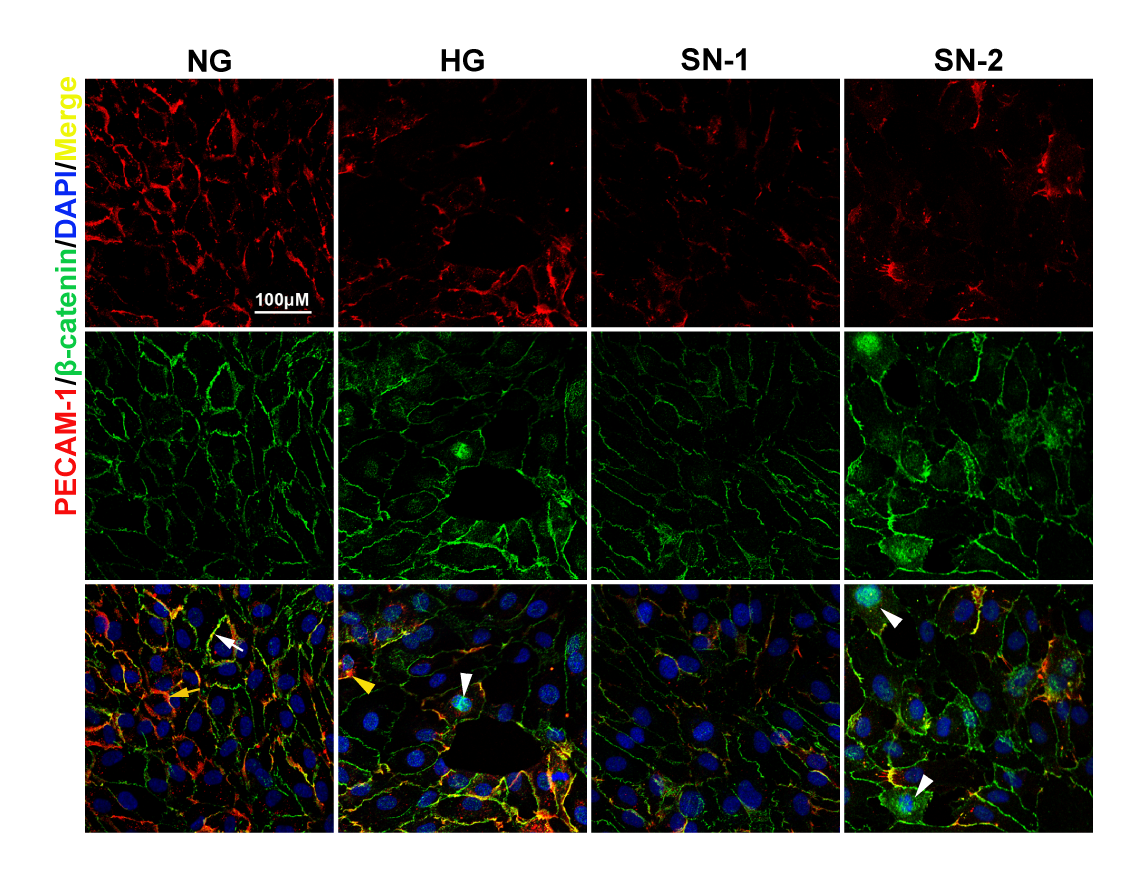


**Figure S5 SNs induces the alteration of PECAM-1 and β-catenin in localization**

Localizations of PECAM-1 and β-catenin were observed after PECAM-1 siRNA treatment by confocal microscopy method under HG and SNs conditions, respectively. White arrows: the β-catenin in membrane; Yellow arrow: the PECAM-1 in membrane; White arrow heads: the β-catenin in the nucleus; Yellow arrow heads: the colocation of PECAM-1 and β-catenin around the nucleus.


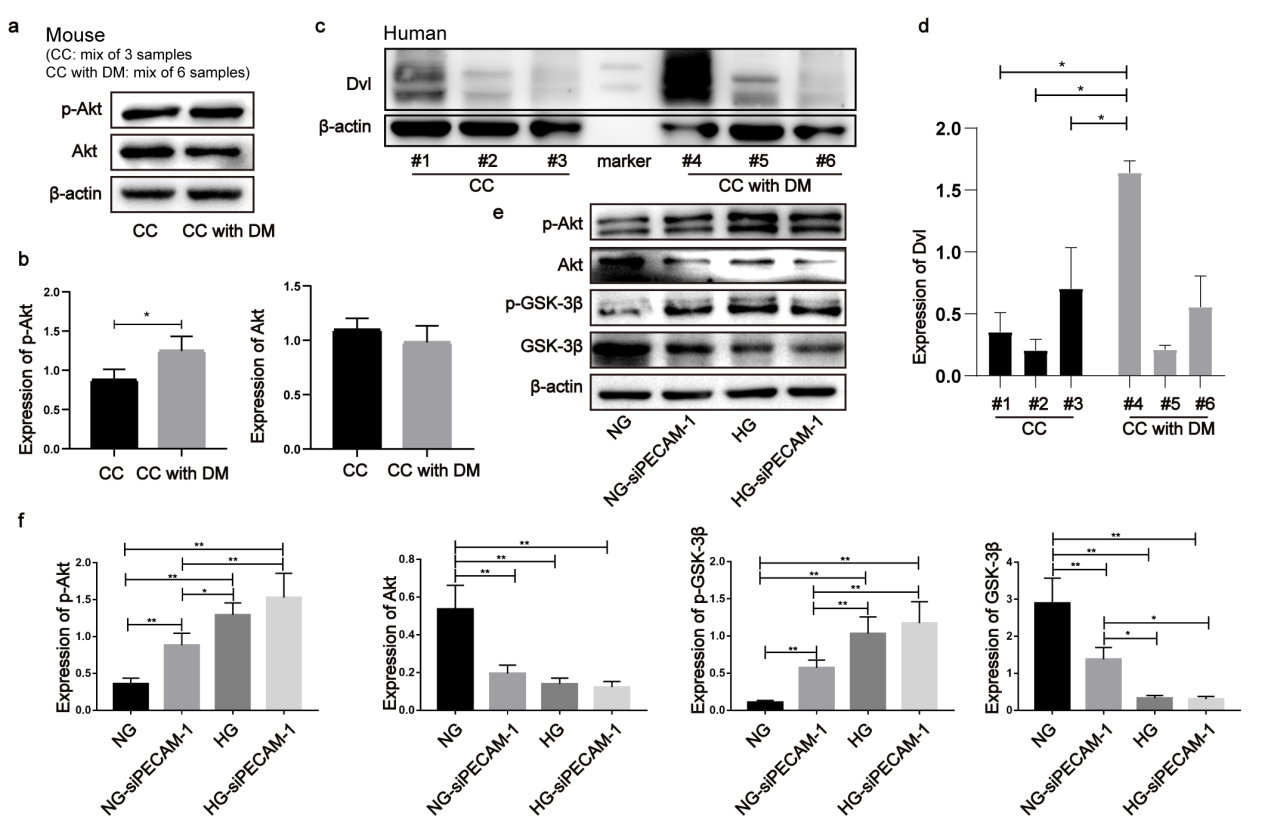


**Figure S6 Expressions of Akt, GSK-3β and Dvl were detected by western blotting**

**a, b** Total and phosphorylated Akt were evaluated in 3-6 mixed samples of CC with and without DM, respectively. **c, d** Dvl was detected in cancerous tissues of CC with and without DM. **e, f** Total and phosphorylated Akt and GSK-3β were assessed after PECAM-1 knocking down. The samples derive from the same experiment and that blots were processed in parallel. Data are means ± S.E.M. and analyzed using one-way ANOVA followed by Tukey’s multiple comparisons test. **p*<0.05, ***p*<0.01.

**Table S1 Primary antibodies in this study**

All of primary antibodies and their working concentrations in this study were listed in supplementary table 1.

**
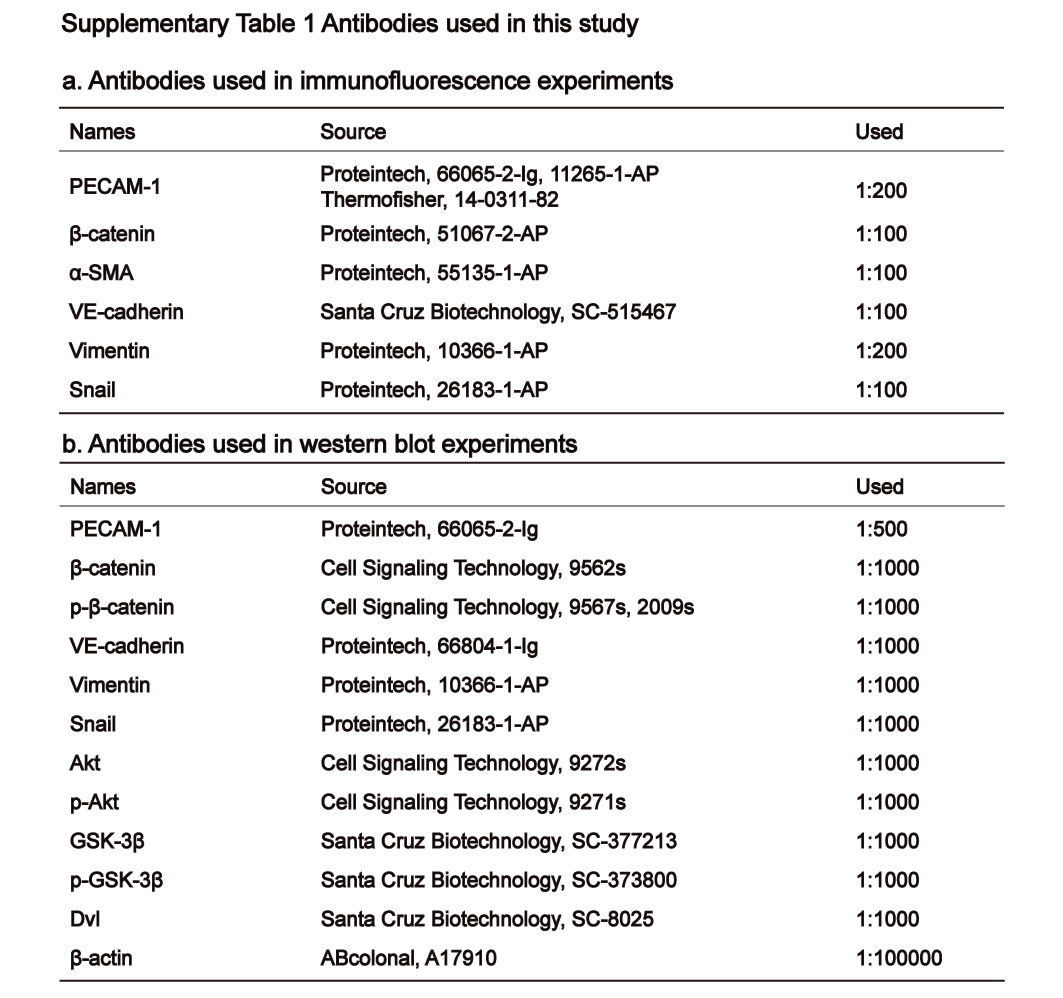
**

**Table S2 Sequences of siRNA in this study**

**
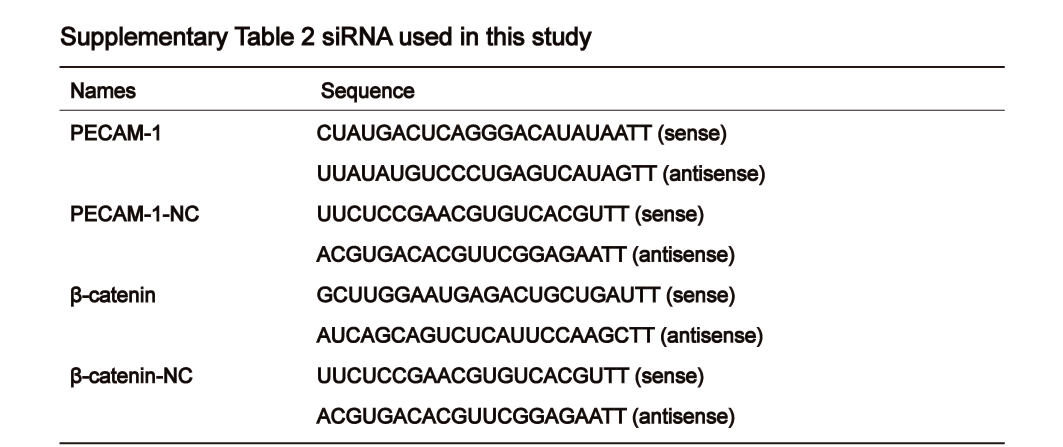
**

**Table S3 Association between clinicopathologic features and DM duration** **in** **CC patients with DM**

**
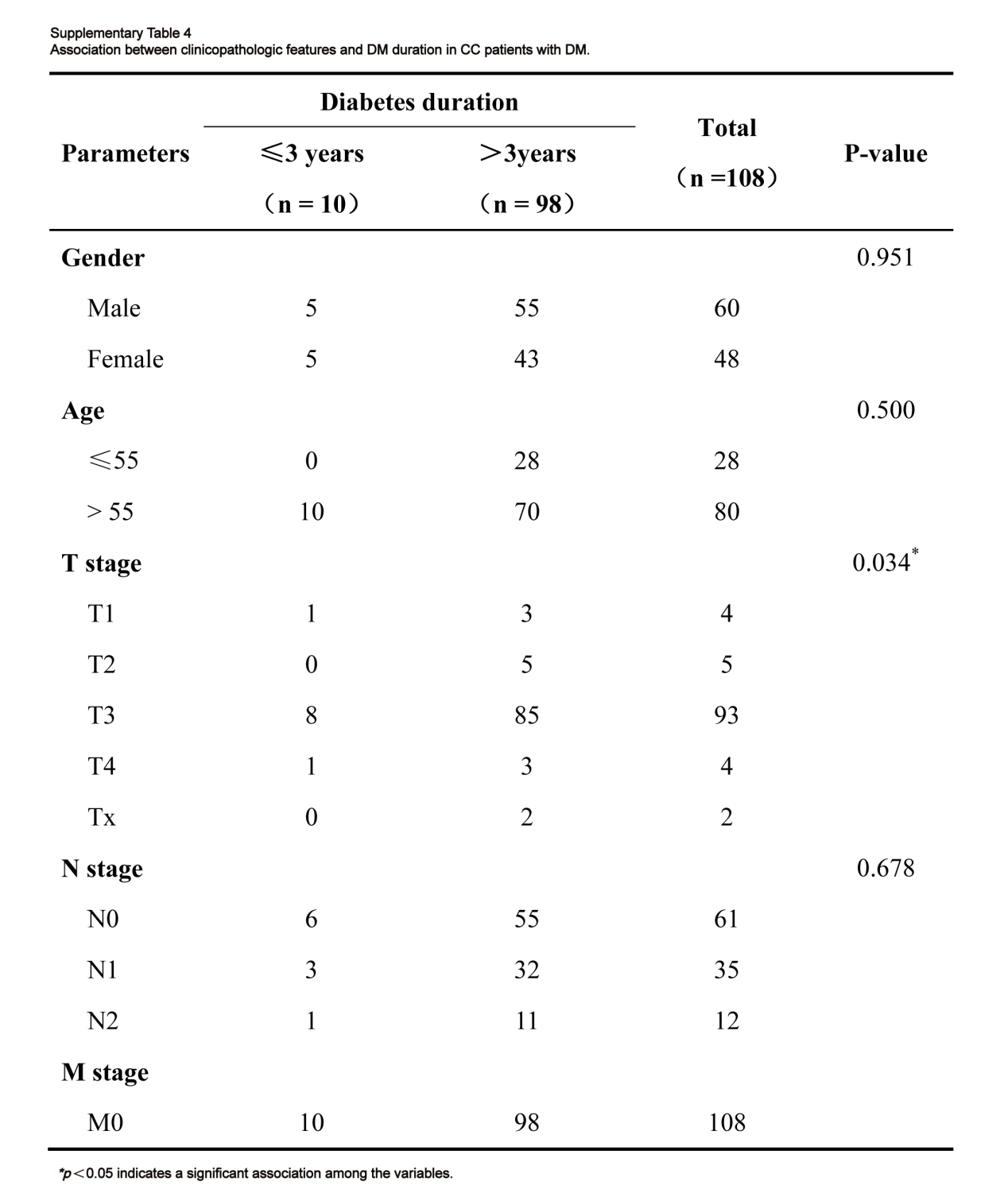
**

Data showed that the group with long DM duration (> 3 years) has a worse TNM stage in stage T than the group with short DM duration in CC patients with DM by one-way ANOVA with Tukey’s multiple comparisons test.
